# E3 ubiquitin-ligases and their target proteins during the regulation of plant innate immunity

**DOI:** 10.3389/fpls.2014.00042

**Published:** 2014-02-13

**Authors:** Vincent Duplan, Susana Rivas

**Affiliations:** ^1^INRA, Laboratoire des Interactions Plantes-Microorganismes (LIPM), UMR441Castanet-Tolosan, France; ^2^CNRS, Laboratoire des Interactions Plantes-Microorganismes (LIPM), UMR2594Castanet-Tolosan, France

**Keywords:** E3 ubiquitin-ligase, microbial effector, plant immunity, 26S proteasome, ubiquitination

## Abstract

Reversible protein ubiquitination plays a crucial role during the regulation of plant immune signaling. E3 ubiquitin (Ub)-ligase enzymes, which are classified into different families depending on their structural and functional features, confer the specificity of substrate and are the best characterized components of the ubiquitination cascade. E3 Ub-ligases of different families have been shown to be involved in all steps of plant immune responses. Indeed, they have been involved in the first steps of pathogen perception, as they appear to modulate perception of pathogen-associated molecular patterns by pattern-recognition receptors at the plasma membrane and to regulate the accumulation of nucleotide-binding leucine-rich repeat-type intracellular immune receptors. In addition, E3 Ub-ligase proteins are also involved in the regulation of the signaling responses downstream of pathogen perception through targeting vesicle trafficking components or nuclear transcription factors, for instance. Finally, we also discuss the case of microbial effector proteins that are able to target host E3 Ub-ligases, or to act themselves as E3 Ub-ligases, in their attempt to subvert the host proteasome to promote disease.

## INTRODUCTION

Reversible protein conjugation with ubiquitin (Ub), or ubiquitination, is a key regulatory mechanism that controls a variety of cellular processes in eukaryotic cells, including DNA repair, gene transcription, protein activation or receptor trafficking, although the best characterized function of Ub involves selective protein degradation through the 26S proteasome (Vierstra, 2009). Ub becomes covalently attached to lysine residues of intracellular targets *via* an ATP-dependent reaction cascade that involves the sequential action of three enzymes: E1 (Ub-activating), E2 (Ub-conjugating), and E3 (Ub-ligase). The importance of the Ub-related pathway is underlined by the finding that the *Arabidopsis *genome encodes more than 1600 genes (> 6% of the total genome) involved in Ub/26S proteasome system (UPS)-related functions. Most of these genes (> 1400) encode putative E3 Ub-ligases (Mazzucotelli et al., 2006). E3 proteins are classified into four main subfamilies depending on their structural features and mechanism of action: HECT (Homologous to E6-associated protein C-Terminus), RING (Really Interesting New Gene), U-Box and CRL (Cullin-RING Ligases; Vierstra, 2009). HECT proteins form an Ub-E3 intermediate before transfer of Ub to the substrate (Downes et al., 2003). RING and U-box proteins are structurally related single polypeptides that, respectively, use zinc chelation and hydrogen bonds/salt bridges to transfer Ub from the Ub-E2 intermediate to the target (Stone et al., 2005; Yee and Goring, 2009). Ub-ligases containing a RING domain can act independently or as part of a multisubunit CRL complex such as the SCF (Skp1, Cullin, F-box)-type ligase. In this complex, substrate recognition is provided by the F-box protein, whereas the RING protein binds to the E2 (Hua and Vierstra, 2011). By contrast, some RING-type Ub-ligases act independently and determine substrate specificity allowing the interaction between the E2 and the target protein by tethering them in close proximity (Vierstra, 2009).

Plants have developed a multi-layered defense system to ensure their survival in a microbe-rich environment. A first line of pathogen detection is activated after recognition of highly conserved PAMPs/MAMPs (pathogen-/microbe-associated molecular patterns) by specific plant PRRs (pattern-recognition receptors), leading to a form of basal resistance called PTI (PAMP-triggered immunity; Jones and Dangl, 2006). Thriving pathogens evolved to secrete virulence effectors that inactivate crucial PTI regulators thereby counteracting plant defenses. In turn, plants gained the ability to recognize these effectors through resistance (R) proteins, for the most part intracellular NB-LRR (nucleotide-binding-leucine-rich repeat) immune sensors, that lead to a more efficient form of resistance called ETI (effector-triggered immunity; Jones and Dangl, 2006). This specific resistance is frequently associated to development of the hypersensitive response (HR), a form of programmed cell death at the infection site that prevents pathogen spreading (Coll et al., 2011).

**FIGURE 1:**
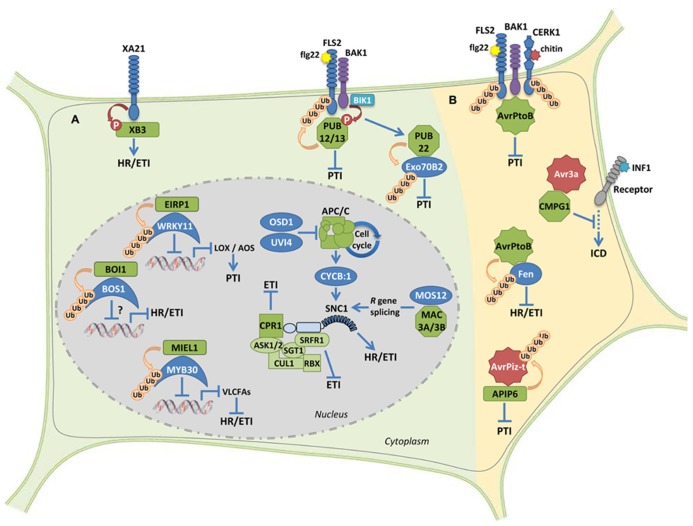
** Ub-ligase proteins during the regulation of plant immune signaling. (A)** Examples of positive and negative regulatory roles on plant immunity by host E3 Ub-ligases. The RING-type protein XB3 is required for the accumulation of the receptor protein XA21 and promotes downstream HR and defense responses. U-box proteins PUB12 and PUB13 attenuate flg22-mediated PTI signaling. The U-box protein PUB22 targets the exocyst complex subunit Exo70B2, which leads to attenuation of PTI signaling. Accumulation of the intracellular immune receptor SNC1 appears to be controlled by the SCF complex through the interaction with both the F-box CPR1 and the SGT1-interacting protein SRFR1. Modulation of *SNC1* levels by the multisubunit cell cycle-related E3 ligase APC/C or the U-box proteins MAC3A and MAC3B provide additional molecular mechanisms to control immunity. The interaction between the RING-type E3 Ub-ligase EIRP1, and the TF WRK11 results in suppressed transcriptional activation of WRKY11 target genes* AOS* and *LOX1*, thereby enhancing PTI. The RING E3 ligase MIEL1 interacts with the MYB TF MYB30, which results in weakened transcriptional activation of VLCFA-related genes and therefore suppressed HR and defense responses. The RING protein BOI1 interacts with and ubiquitinates the MYB TF BOS1 that is required for pathogen resistance. However, the effect of BOI1 on BOS1 accumulation and transcriptional activation remains unknown. **(B)** Microbial effector proteins acting as or interacting with E3 ligase proteins in the host. The bacterial effector AvrPtoB is able to suppress both PTI and ETI signaling though its respective interaction with FLS2/CERK1 PRRs and the cytoplasmic kinase Fen. The oomycete effector AVR3a suppresses INF1-induced cell death by interacting with and stabilizing the host U-box E3 ligase CMPG1. The fungal effector protein AvrPiz-t targets the RING E3 ligase APIP6, which in turn is able to ubiquitinate AvrPiz-t, resulting in suppressed PTI. RING-, F-box- and U-box-type E3 Ub-ligases are, respectively, represented by rectangles, squares, and octagons. All proteins displaying E3 ligase activity are represented in green. Effectors are represented by stars. See the text for details.

In plants, the UPS pathway, and more particularly E3 Ub-ligase proteins, have been shown to be involved in responses to a variety of stimuli (Vierstra, 2009; Robert-Seilaniantz et al., 2011). Since the finding that the SCF complex-interacting protein SGT1 (Suppressor of G2 allele of skp1) is an essential component of *R* gene-triggered disease resistance provided a first connection between the UPS and plant immune signaling (Azevedo et al., 2002), evidence that E3 Ub-ligase proteins act as regulators of plant immunity has increasingly accumulated (Trujillo and Shirasu, 2010; Marino et al., 2012). Indeed, modulation of the expression of E3 Ub-ligase-encoding genes has been reported following elicitor treatment or inoculation with different pathogens. Moreover, misregulation of E3 Ub-ligase gene expression, using overexpressing, RNA interference (RNAi) and/or mutant lines, results in modulation of plant defense responses following pathogen inoculation [reviewed in (Marino et al., 2012)]. Therefore, it has become increasingly evident that plant E3 Ub-ligase proteins play important roles in the regulation of immune signaling, although the proteins targeted by Ub-ligases are only known in a limited number of cases, and our current knowledge of the involved molecular mechanisms is thus only partial. Here we review positive and negative roles played by E3 Ub-ligases during the regulation of various steps of plant immunity, from pathogen recognition to downstream signaling during both PTI and ETI responses. Due to space limitations, we focus on recent reports about E3 Ub-ligases for which a target protein has been identified during the plant response to bacterial or fungal pathogens, since these particular examples provide insight into the cellular processes involved in regulation of immune signaling. For an overview on UPS-related pathways in response to viral infection we refer the reader to a recent review (Alcaide-Loridan and Jupin, 2012). We also discuss the case of microbial effectors that, to promote disease, either target host E3 Ub-ligases or act as Ub-ligases inside plant cells (**Figure 1**).

## PLANT E3 UB-LIGASES INVOLVED IN REGULATION OF PATHOGEN PERCEPTION

Several E3 ligase proteins have been identified as modulators of the first steps of pathogen recognition by plant cells, as they appear to be able to target both PRR and NB-LRR proteins in order to prevent unnecessary activation of defense signaling. In rice (*Oryza sativa*), the RING-type E3 Ub-ligase XB3 interacts with the receptor-like kinase (RLK) protein XA21, which confers resistance to bacterial blight caused by *Xanthomonas oryzae* pv. *oryzae* (*Xoo*; Wang et al., 2006). XB3 has been shown to be required for XA21 accumulation and XA21-mediated resistance to *Xoo*, suggesting that it most likely targets a protein that modulates accumulation of XA21**(Wang et al., 2006). XA21 is able to phosphorylate XB3 but the molecular mechanism underlining activation of defense responses by XB3 remains to be elucidated. Overexpression of members of the XB3 family from rice, *Arabidopsis* and citrus in *Nicotiana benthamiana* induces cell death and this effect is dependent on XB3 catalytic activity, suggesting an evolutionarily conserved role for the XB3 protein family in regulating plant programmed cell death (Huang et al., 2013).

In *Arabidopsis*, the U-box E3 ligases Plant U-Box12 (PUB12) and PUB13 have been involved in attenuation of PTI responses triggered by perception of flagellin, or its active peptide derivative flg22, by the PRRs Flagellin Sensing2 (FLS2) and its co-receptor BAK1. In response to flg22, PUB12 and PUB13 form a BAK1-dependent complex with FLS2 and are able to polyubiquitinate FLS2, but not BAK1 (Lu et al., 2011). BAK1 phosphorylates PUB12 and PUB13 and this phosphorylation is enhanced by flg22 and by the FLS2/BAK1-associated kinase BIK1 (Lu et al., 2011). flg22-dependent signaling is enhanced in *pub12* or *pub13* mutant plants and, in agreement with PUB12 and PUB13 promoting FLS2 degradation, *pub12 pub13* double mutant plants displayed increased resistance to bacterial infection. These data are consistent with the fact that FLS2 undergoes flg22-induced endocytosis and subsequent degradation (Robatzek et al., 2006; Gohre et al., 2008; Beck et al., 2012; Choi et al., 2013). However, whether stabilization of FLS2 in *pub12pub13* plants reflects FLS2 accumulation at the plasma membrane or within an endosomal compartment remains to be determined. A recent report showed that FLS2 degradation occurs in a flg22 time- and dose-dependent manner, which may play a significant role in turning over ligand-occupied FLS2, but the role of PUB12 and PUB13 in this process was not determined in this study (Smith et al., 2013).

Similar to PRR proteins, intracellular NB-LRR immune receptors are also targeted by E3 Ub-ligases, which appear to control R protein accumulation at multiple levels. First, in *Arabidopsis*, the F-box motif E3 Ub-ligase CPR1 interacts with and down-regulates the accumulation of the NB-LRR R proteins Suppressor of *npr1-1* Constitutive1 (SNC1) and Resistant to *P. syringae2* (RPS2), resulting in attenuation of immune signaling (Cheng et al., 2011; Gou et al., 2012). Second, accumulation of SNC1 and RPS4, an additional NB-LRR immune receptor, are also negatively regulated by Suppressor of *rps4-RLD1* (SRFR1), a tetratricopeptide repeat protein (Kim et al., 2010; Li et al., 2010). Since (i) SNC1 levels increased in CPR1-overexpressing plants treated with the proteasome inhibitor MG132 (Gou et al., 2012); (ii) SRFR1 interacts with SGT1; and (iii) increased SNC1 and RPS4 accumulation was also observed in *sgt1* mutant plants (Li et al., 2010), stability of SNC1 and RPS4 is likely regulated by SRFR1 through SGT1 interaction with the SCF complex, revealing an additional molecular mechanism to prevent autoimmnunity. Third, mutation of *MOS12 *(*modifier of snc1-12*), that encodes an *Arabidopsis* Arg-rich protein homologous to human cyclin L, resulted in altered *SNC1* and *RPS4 *splicing patterns and protein levels (Xu et al., 2012). Interestingly, MOS12 interacts with the nuclear U-Box E3 ligases MAC3A and MAC3B, which are required for full R protein-mediated resistance, suggesting that MOS12, MAC3A, and MAC3B contribute to the fine-tuning of *R* gene expression, in a process that appears to be critical for directing appropriate defense outputs (Monaghan et al., 2009; Xu et al., 2012). Finally, a recent report showed an intriguing link between cell cycle regulation and defense signaling (Bao et al., 2013). Omission of the Second Division (OSD1) and its homolog UV-B-Insensitive 4 (UVI4) are two negative regulators of the multisubunit E3 Ub-ligase APC/C (anaphase-promoting complex/cyclosome) that regulates cell cycle progression in *Arabidopsis*. Overexpression of either OSD1 or UVI4 leads to downregulation of APC/C activity, overaccumulation of the APC/C degradation target CYCB1;1, upregulation of several *R* genes, including *SNC1*, and spontaneous cell death and enhanced disease resistance to virulent bacteria (Bao et al., 2013). These data provide further evidence of the intricate control exerted on immune receptor levels in order to regulate defense activation.

## PLANT E3 UB-LIGASES INVOLVED IN REGULATION OF DEFENSE-RELATED SIGNALING

Pathogen perception by PRRs stimulates a cascade of signaling events including changes in ion fluxes across the plasma membrane, production of reactive oxygen species (ROS), induction of mitogen-activated protein kinases (MAPKs), modulation of host gene transcription and callose deposition at the plant cell wall. Amplitude and duration of these signaling responses must be tightly regulated to ensure an appropriate response. In addition to their role in internalization or degradation of receptors to attenuate downstream signaling, E3 Ub-ligase proteins also regulate the accumulation of plant components involved in defense-related signaling.

Similar to PUB12 and PUB13, PUB22 acts in concert with PUB23 and PUB24 to negatively regulate PTI responses in *Arabidopsis* (Trujillo et al., 2008). Following elicitation with various PAMPs, *pub22/pub23/pub24* triple mutants displayed enhanced early signaling responses, indicating that these three PUB proteins target components involved in defense signaling triggered by different PRRs (Trujillo et al., 2008). Indeed, the exocyst complex subunit Exo70B2 that is involved in vesicle tethering during exocytosis, has been identified as a cellular target of PUB22 (Stegmann et al., 2012). PUB22 is stabilized in response to flg22 treatment (potentially by inhibition of its autocatalytic ubiquitination activity), leading to Exo70B2 ubiquitination and proteasomal degradation. Exo70B2 is required for both immediate (ROS production, MAPK activation) and later responses (PTI marker gene expression, root growth inhibition) triggered by several PAMPs, indicative of a role in signaling. Indeed, *exo70B2* mutant plants displayed enhanced susceptibility to pathogens (Stegmann et al., 2012). Together, these data suggest a mechanism by which Exo70B2 levels are regulated by quick changes in PUB22 turnover in response to PAMPs and identify a first component of vesicle trafficking required for regulation of plant PTI signaling. In view of these data, it has been proposed that Exo70B2 may contribute to recycling of plasma membrane proteins involved in PAMP-triggered signaling, including NADPH oxidases, ion channels or RLKs such as FLS2. Exo70B2 degradation by PUB22 would thus attenuate the recycling pathway redirecting positive signaling components into the vacuolar degradation pathway and downregulating signaling (Stegmann et al., 2012). As previously discussed, PUB12/PUB13-mediated ubiquitination of FLS2 is expected to modulate its intracellular trafficking (Lu et al., 2011). Since degradation of integral membrane proteins is mediated by the vacuole, signal attenuation is probably simultaneously regulated at various levels of vesicle trafficking.

In Chinese wild grapevine (*Vitis pseudoreticulata*), EIRP1 is an active E3 Ub-ligase whose RING domain is necessary for its activity and also mediates the interaction with the WRKY nuclear transcription factor (TF) VpWRKY11 (Yu et al., 2013). Similar to *EIRP1*, expression of *VpWRKY11* was rapidly induced following fungal infection*.* VpWRKY11 activated the expression of *AOS *(*Allene Oxide Synthase*) and *LOX2 *(*Lipoxygenase2*), two JA-responsive genes that function as negative regulators of basal resistance in *Arabidopsis* (Journot-Catalino et al., 2006). Additionally, in agreement with the observation that co-expression with EIRP1 results in proteasomal degradation of VpWRKY11, *AOS* and *LOX2* expression was, respectively, repressed and induced in EIRP1-overexpressing and RNAi plants. Moreover, EIRP1 overexpression in *Arabidopsis* conferred enhanced resistance to fungal and bacterial pathogens, which correlated with reduced expression of *WRKY11, AOS,* and *LOX2 *(Yu et al., 2013)*. *Together, these data identify a RING-type Ub-ligase that plays a positive role in activation of resistance by targeting a TF that acts as a negative regulator of plant defenses.

In contrast, the *Arabidopsis* RING-type Ub-ligase MIEL1 acts as a negative regulator of plant resistance (Marino et al., 2013). Indeed, MIEL1 interacts with the MYB TF MYB30, which activates plant defense responses by up-regulating the expression of genes involved in the production of very long chain fatty acids (VLCFAs; Raffaele et al., 2008). The MYB30-MIEL1 nuclear interaction leads to MYB30 proteasomal degradation, reduced expression of VLCFA-related MYB30 target gene expression and, therefore, attenuation of plant immune responses (Marino et al., 2013). *MIEL1* expression is rapidly repressed in inoculated cells, suggesting that (i) in the absence of the pathogen, MIEL1 may negatively regulate plant defense activation through degradation of MYB30 and that (ii) after pathogen inoculation, repression of *MIEL1* expression may release MYB30 negative regulation, triggering defense (Marino et al., 2013).

BOI1 is an additional nuclear RING-type Ub-ligase that interacts with and ubiquitinates the MYB TF BOS1, which confers resistance to several pathogens in *Arabidopsis* (Mengiste et al., 2003; Luo et al., 2010). This finding suggests that BOS1 may be a target of BOI1. However, no effect of BOI1 on BOS1 transcriptional activity has been reported and both *bos1* mutant and BOI1 RNAi *Arabidopsis* plants (in which the BOS1 protein is expected to accumulate) display enhanced susceptibility to fungal infection (Luo et al., 2010). Therefore, whether BOI1 is able to directly regulate BOS1 protein accumulation remains to be determined.

## MANIPULATION OF HOST E3 UB-LIGASE PROTEINS BY MICROBIAL EFFECTORS

The important role played by E3 Ub-ligases during the establishment of plant immune responses to pathogen attack is highlighted by the discovery of microbial effector proteins that evolved the ability to interfere with these host UPS components to promote disease. For example, the AvrPiz-t effector from the rice blast fungus *Magnaporthe oryzae* is translocated into rice cells, where it is able to mediate suppression of PAMP-induced ROS production, inducing susceptibility to *M. oryzae *(Park et al., 2012). AvrPiz-t appears to inhibit the Ub-ligase activity of APIP6, a rice RING-type Ub-ligase that is also able to ubiquitinate AvrPiz-t *in vitro* (Park et al., 2012). Interestingly, AvrPiz-t and APIP6 are both degraded when transiently coexpressed in *N. benthamiana*. Since APIP6 positively regulates flg22-induced ROS generation, induction of defense-related gene expression, and rice resistance to *M. oryzae*, targeting of APIP6 by AvrPiz-t results in suppression of rice PTI responses (Park et al., 2012).

The effector AVR3a from the oomycete *Phytophthora infestans* prevents development of cell death induced by *P. infestans* elicitin INF1. The finding that AVR3a targets and stabilizes the U-box-type Ub-ligase CMPG1 revealed the molecular mechanism behind AVR3a negative regulation of ICD (INF1-triggered Cell Death; Bos et al., 2010). CMPG1 Ub-ligase activity is required for ICD as well as for cell death following elicitor perception at the plasma membrane (Gonzalez-Lamothe et al., 2006; Gilroy et al., 2011). Considering that AVR3a is essential for *P. infestans* virulence, stabilization of CMPG1 by AVR3a suggests that this effector is able to suppress ICD during the biotrophic phase of infection by modifying CMPG1 activity, impeding normal proteasomal degradation of both CMPG1 and its host targets (Bos et al., 2010).

In addition to microbial effectors that are able to target host E3 Ub-ligase proteins, examples of effectors that present E3 Ub-ligase-related domains have also been reported in a diversity of pathogenic microbes including bacteria, fungi, oomycetes, viruses and nematodes (Marino et al., 2012). The best characterized example of microbial E3 ligases is the AvrPtoB effector from *Pseudomonas syringae* that presents a C-terminal domain with remarkable structural homology with RING- and U-box-type Ub-ligases (Janjusevic et al., 2006). AvrPtoB is a modular effector able to suppress PTI signaling. Indeed, AvrPtoB N-terminal domain is able to interact with the PRRs FLS2 and the chitin receptor CERK1, whereas its C-terminal Ub-ligase domain mediates PRR proteasomal degradation (Gohre et al., 2008; Gimenez-Ibanez et al., 2009). AvrPtoB is additionally able to target the co-receptor protein BAK1 (Shan et al., 2008) and to interfere with MAPK activation downstream of FLS2 (He et al., 2006), although independently of AvrPtoB Ub-ligase activity. Remarkably, AvrPtoB is also able to suppress ETI signaling. Indeed, AvrPtoB interacts with and mediates proteasomal degradation of Fen, a tomato protein kinase that activates plant immunity in response to *P. syringae* carrying Ub-ligase deficient forms of AvrPtoB, as well as cell death responses when overexpressed in *N. benthamiana* (Rosebrock et al., 2007).

## CONCLUSIONS AND PERSPECTIVES

Evidence of the involvement of the UPS pathway in the regulation of plant immunity is rapidly mounting. E3 Ub-ligase proteins are the best characterized UPS components playing a role in immune signaling at multiple levels. Nevertheless, based on induction of their expression following elicitation, E1 and E2 enzymes have also been suggested to contribute to plant disease resistance although the molecular mechanisms by which they regulate this process remain poorly characterized (Marino et al., 2012). Interestingly, a recent report provides a first example of the direct involvement of Ub-conjugating proteins in the regulation of plant immunity. In tomato, Fen-interacting protein 3 (Fni3) is a homolog of the *Arabidopsis* E2 enzyme Ubc13. Through interaction with its cofactor *S. lycoperiscum* Uev (Suv), which is an inactive Ub-conjugating enzyme variant, Fni3 catalyzes Lys-63-linked ubiquitination, a non-proteolytic regulatory signal (Mural et al., 2013). Fni3 interacts with Fen but does not affect Fen stability. Fni3 Ub-conjugating activity and interaction with Fen are required for cell death triggered by Fen overexpression in *N. benthamiana* and by several R protein/effector pairs (Mural et al., 2013). Together these results suggest that, in addition to conventional Lys-48-linked ubiquitination that mainly serves as a signal for proteasomal degradation of substrate proteins, other ubiquitination forms are important regulators of plant immune signaling. Consistent with this idea, Lys-63-linked ubiquitination has also been shown to be a target of manipulation by plant pathogens, as the PthA effector from the bacterial pathogen *Xanthomonas axonopodis* pv. *citri* is able to target Ubc13 and Uev in citrus (Domingues et al., 2010).

In conclusion, further characterization of additional UPS components as well as of the distinct fates of ubiquitination targets should contribute to dissecting the complex regulation of plant immune signaling by the UPS.

## Conflict of Interest Statement

The authors declare that the research was conducted in the absence of any commercial or financial relationships that could be construed as a potential conflict of interest.
